# Strategies for the Development of Conotoxins as New Therapeutic Leads

**DOI:** 10.3390/md11072293

**Published:** 2013-06-28

**Authors:** Ryan M. Brady, Jonathan B. Baell, Raymond S. Norton

**Affiliations:** Monash Institute of Pharmaceutical Science, Monash University, 381 Royal Parade, Parkville 3052, Australia; E-Mails: ryan.brady@monash.edu (R.M.B.); jonathan.baell@monash.edu (J.B.B.)

**Keywords:** peptide toxin, peptidomimetic, ion channel, pain, cone snail

## Abstract

Peptide toxins typically bind to their target ion channels or receptors with high potency and selectivity, making them attractive leads for therapeutic development. In some cases the native peptide as it is found in the venom from which it originates can be used directly, but in many instances it is desirable to truncate and/or stabilize the peptide to improve its therapeutic properties. A complementary strategy is to display the key residues that make up the pharmacophore of the peptide toxin on a non-peptidic scaffold, thereby creating a peptidomimetic. This review exemplifies these approaches with peptide toxins from marine organisms, with a particular focus on conotoxins.

## 1. Introduction

The marine environment has proven to be a valuable source of interesting and unusual natural products with a diverse range of biological activities. Of particular interest are marine snails belonging to the genus *Conus*, which contains an estimated 700 species, each possessing a unique cocktail of pharmacologically active peptides within its venom [1,2,3]. These marine snails have evolved into efficient predators, using their venom to hunt and paralyze worms, molluscs or fish. Conotoxins, isolated from the venom ducts of cone snails, constitute a large family of small, disulfide-rich peptides that have evolved to target a range of ion channels and receptors throughout the nervous system, usually with high potency and selectivity [4,5,6,7]. As such, many of these conotoxins have been used to gain further information about their target at the pharmacological, physiological or structural level [8,9,10,11]. They are relatively small peptides, typically eight to thirty amino acid residues in length that have been divided into different structural and pharmacological classes. A nomenclature for the conotoxins classifies the peptides according to the source, cysteine framework and biological target [12]. The more recent availability of nucleic acid sequences from cDNA and transcriptomics analyses is enabling systematic classification into superfamilies on the basis of pre- and pro-peptide sequences [13,14].

Conotoxins serve not only as valuable pharmacological tools but potential drug candidates. While several conotoxins have advanced to clinical trials [15,16,17], ω-conotoxin MVIIA (ziconotide) was the first to be approved by the FDA for therapeutic use in humans [18]. Marketed as Prialt^®^, it possesses potent and selective N-type calcium channel activity and is used to treat patients suffering from severe chronic pain [19]. Although Prialt^®^ represents a major milestone for conotoxins, its use is limited to intrathecal administration. Nonetheless, it highlights the potential of neurotoxic peptides as starting points for the development of therapeutics. Despite their desirable biological activities, peptides generally have several limitations that have restricted their progression as drug candidates, amongst which are short circulating half-life, poor proteolytic stability, and low oral bioavailability [20,21]. The challenge remains to capture the favorable bioactive properties of peptide toxins within drug-like molecules that can be administered in the clinic. This review summarizes current strategies for the development of conotoxins and their mimetics as leads for novel therapeutics.

## 2. Peptidomimetics

One approach to developing bioactive small molecules is the rational design of organic scaffolds that topographically mimic the key binding elements (pharmacophore) of the native peptide. By utilizing a non-peptidic scaffold, peptidomimetics can potentially circumvent the inherent limitations of peptides, notably stability and bioavailability [22]. The design of peptidomimetics can be classified into three distinct classes [23]: Type I involves replacement of amide bond with isosteres that reproduce the peptide conformation. Type II mimetics are defined as small molecules that bind to a protein but do not structurally mimic the native interaction. Type III mimetics are non-peptidic molecules designed to mimic the spatial arrangement of key amino acid side chains in the peptide. In each case, there are many examples of successful design of peptidomimetics that have retained biological activity and significantly improved pharmacokinetic properties [24,25,26]. Key to designing such peptidomimetics is a knowledge of both the peptide pharmacophore and the peptide structure.

**Figure 1:**
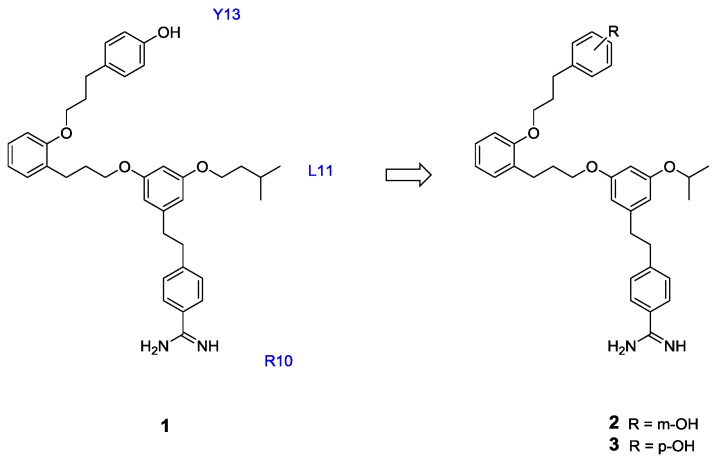
The dendroid scaffold **1** designed to mimic Arg10, Leu11 and Tyr13 in ω-conotoxin MVIIA, and subsequent analogues **2** and **3**, which further explored Leu11 and Try13 side chain mimics [33].

The peptidomimetic strategy has been applied to several members of the conotoxin family. The ω-conotoxins, which block N-type voltage-gated calcium channels (Ca_V_2.2), have been investigated extensively because of their promising analgesic activity [27,28]. Menzler *et al.* [29] have described peptidomimetics of ω-conotoxin MVIIA, a 25-residue peptide with potent and selective N-type calcium channel-blocking activity [30,31]. The peptidomimetics were based on a “dendroid” approach, where three amino acid side chains were incorporated into a central aromatic core **1** [32,33]. Based on the solution structure of ω-MVIIA, the functionalized side chains attached to the dendroid core displayed appropriate spatial mimicry of the Tyr13, Leu11 and Arg10 residues. The designed mimetic displayed weak N-type calcium channel activity, although further analogues of **1** were designed and found to have improved voltage-gated calcium channel (VGCC) activity (Figure 1).

**Figure 2:**
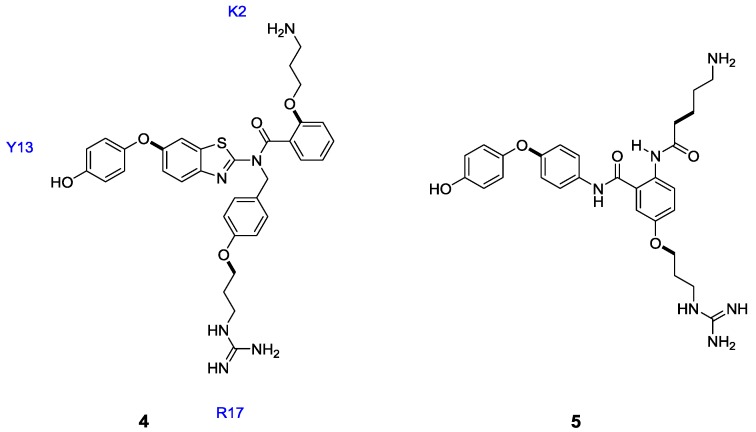
Type III peptidomimetics of ω-GVIA using the *in silico* *de novo* design methodology. A benzothiazole **4** and an anthranilamide **5** scaffold were designed to mimic the Lys7, Try13 and Arg17 side chains in the native peptide [38].

Type III peptidomimetics of ω-conotoxin GVIA have been conceived via an interactive *de novo* design. ω-GVIA, a 27-residue peptide produced by *Conus geographus*, has been shown to potently block the neuronal voltage-gated N-type calcium channel [34,35]. In the *de novo* design approach, novel scaffolds are interactively designed *in silico* to overlay with the C_α_–C_β_ bond vectors of important amino acid residues. Guided by previous structure-function studies [36,37], the bond vectors of Arg17, Try13 and Lys2 were mimicked with two different scaffolds; a benzothiazole **4** and an anthranilimide **5** (Figure 2) [38]. The benzothiazole **4** was found to block rat VGCC (Ca_V_2.2) with an IC_50_ of 98 μM, measured as a response to sympathetic nerve-mediated contraction of rat *vas deferens* [37]. This level of activity could be considered moderate, yet such a result allowed for potential optimization of the interactions of **4** with the channel, given that analogues could be readily synthesized.

**Figure 3:**
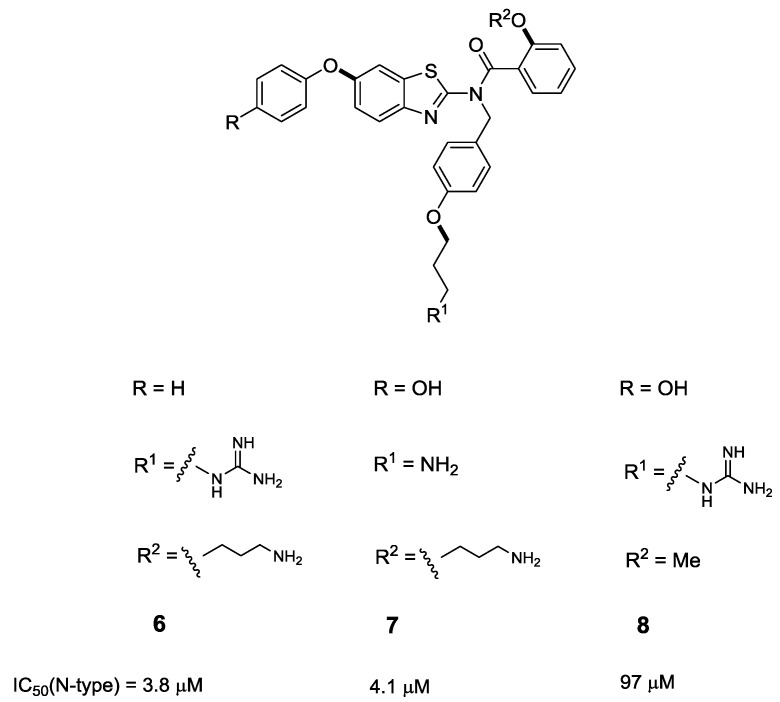
Modifications of the benzothiazole scaffold exploring the relative contributions of the side chain functionalities to binding [39].

To this end, Baell and co-workers investigated the relevant contribution of the three side chain mimics to binding affinity by way of competitive radioligand binding assay, where the affinity of each compound for the N-type channel was determined by displacement of ^125^I-labelled ω-GVIA from rat brain membrane [39]. In this assay, the designed mimetic **4**, displayed an IC_50_ of 1.9 μM. The des-hydroxy analogue **6** was found to be two-fold less potent than **4**. Importantly, however, this analogue displayed selectivity for N-type (Ca_V_2.2) *vs.* P/Q-type (Ca_V_2.1) channels. A primary amino group in place of the guanidine moiety also resulted in a two-fold loss in activity (Figure 3, Compound **7**). Replacement of the alkylamine side chain, designed to mimic Lys2, was the most notable deletion in Analogue **8**, essentially abrogating activity. This suggests that the alkyl amino moiety is a substantial contributor to VGCC, highlighting the importance of mimicking the Lys2 residue.

**Figure 4:**
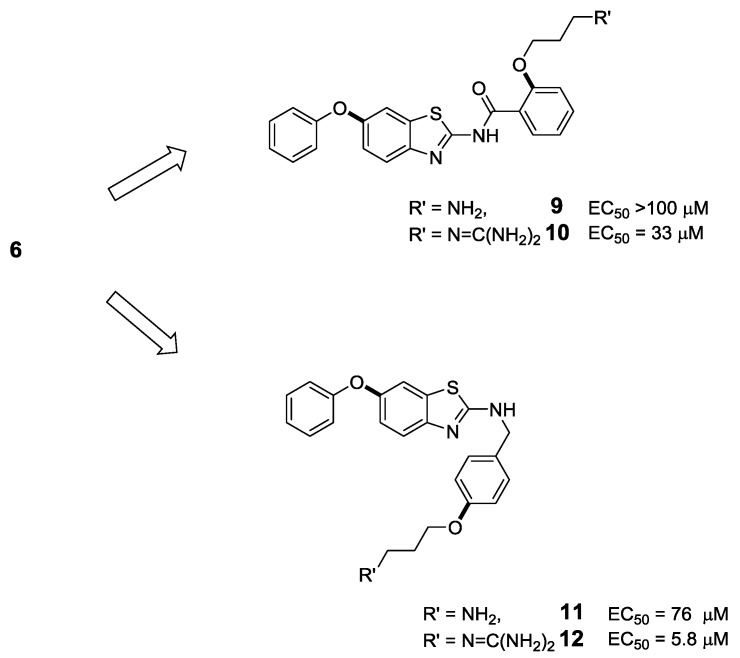
Truncated analogues of the benzothiazole-based ω-GVIA mimetic **6** [40]. In either approach, removal of the guanidine functionality proved detrimental to activity. Compound **12** displayed activity comparable to **6**, highlighting the importance of mimicking the Arg17 side chain and a preferred conformation of the *N*-benzyl moiety.

Further studies into the potential of **6** as a VGCC blocker were carried out by Duggan and co-workers, who explored truncated analogues rationalized by conformation around the *N*-benzyl moiety [40]. These authors hypothesized that rotation around the *N*-benzyl bond could result in two conformations, whereby the Arg17 mimic could orientate above or below the plane of the benzothiazole core. Excising the *N*-benzyl motif, leading to the two amino acid residue mimic **9**, resulted in a loss of binding in a radioligand-binding displacement assay (Figure 4). Guanidinylation of the terminal amine in the truncated Analogue **10** salvaged activity (EC_50_ 33 μM), yet its activity was still 10-fold less than the original Compound **4**. Truncation at the amide bond produced the Tyr13 and Arg17 mimetic **12**, which was, surprisingly, equipotent with the original Compound **4**, having an EC_50_ of 5.8 μM. Removal of the guanidine functionality in **12** led to a loss in VGCC activity, highlighting the importance of the strongly basic functionality (Figure 4, Compound **11**). Whilst this study did not produce a substantial breakthrough compound in terms of bioactivity, its significance lies in the fact that the truncated Analogue **12** retains affinity yet represents a significant reduction in molecular weight (193 g/mol) relative to the original mimetic. It remains to be seen whether these truncated analogues display useful activity in a functional assay. Nonetheless, such truncated molecules could be more readily accessed synthetically and could serve as templates for medicinal chemistry optimization and ultimately discovery of more potent VGCC blockers.

**Figure 5:**
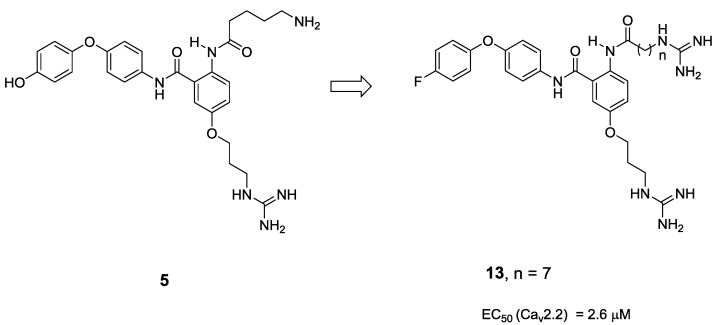
Using the anthranalimide peptidomimetic scaffold **5** as a platform for medicinal chemistry exploration. Replacement of the phenol with a fluorine atom along the introduction of alkyl guanidine’s afforded Compound **13**, possessing an EC_50_ for Ca_v_2.2 of 2.6 μM [41].

Similar success was achieved using the anthranilamide **5** as a ω-GVIA peptidomimetic (Figure 5). The functionalized molecule bearing the Try13, Arg17 and Lys2 side chain mimics blocked Ca_V_2.2 with micromolar affinity in a functional assay (68 μM) [41]. Further structure-activity relationship (SAR) studies surrounding the mimetic focused on the nature of the alkyl side chains and the use of both amino and guanidine groups [42,43]. Variation of the side chain lengths of both the Lys and Arg mimics did not generate any convincing SAR, although varying the terminal functionality on the Lys and Arg side chains did significantly affect activity. The use of amino groups at both of these positions resulted in weak VGCC activity, regardless of the chain length. On the other hand guanidines at both these positions proved to be more active. Furthermore, introduction of a fluoro atom into the tyrosine mimic afforded the most active compound in the study, with an EC_50_ of 2.6 μM for Cav2.2 (Figure 5). It should be noted, however, that calcium channel activity was evaluated via a radioligand-binding displacement assay, in contrast to the functional assay used for Compound **5**, and thus a meaningful comparison is difficult. Unlike the benzothiazole **4**, no further deletion or truncated analogues have been reported. 

**Figure 6:**
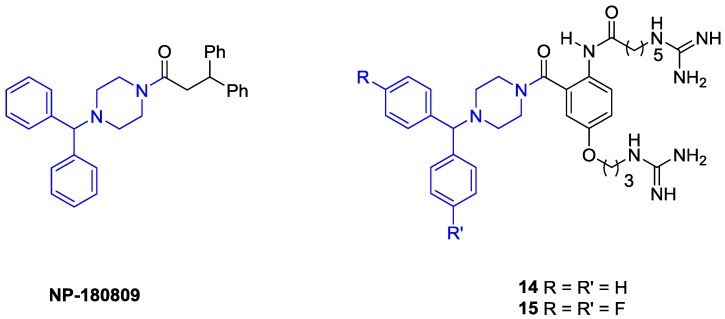
The calcium channel blocker developed by Neuromed, NP-180809 [47], and incorporation of the diphenylmethylpiperazine motif within the anthranilamide peptidomimetic [44].

In a recent study, Tranberg *et al.* [44] designed and synthesized a “hybrid” molecule based on the anthranilimide **10** and a diphenylmethylpiperazine, a common moiety found in calcium channel blockers developed by Neuromed and Abbott laboratories (Figure 6) [45,46,47,48]. The diphenylmethylpiperazine analogues **14** and **15**, which were analogous with the parent anthranilimide, displayed low micromolar EC_50_’s in a ^125^I-ω-GVIA displacement assay. However, both molecules could block functional ion channels in a whole-cell patch clamp assay. Whilst this level of functional activity has not been observed previously with this series of mimetics, the most potent Compound **15** displayed an IC_50_ of 156 μM, which is still several orders of magnitude weaker than ω-GVIA.

**Figure 7:**
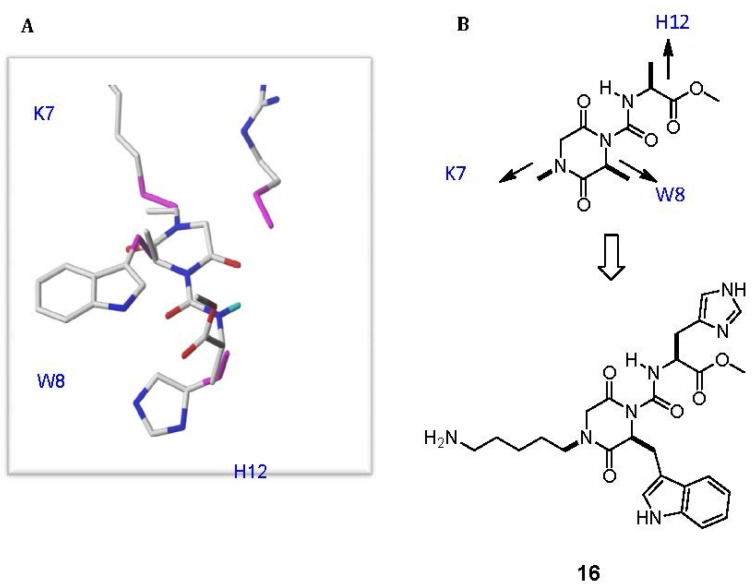
(**A**) *In silico* de novo designed μ-KIIIA peptidomimetic based on a diketopiperazine carboxamide scaffold. (**B**) The functionalized molecule incorporating Lys7, Trp8 and His12 mimetics.

The *de novo* design strategy has also been applied to μ-conotoxin peptidomimetics. Isolated from *Conus kinoshitai*, μ-KIIIA is a 16-residue peptide that blocks neuronal voltage-gated sodium channels (VGSC) and displays potent analgesic activity when administered in mice [49]. As such, μ-KIIIA could serve as a template for the development of novel analgesics. In keeping with other μ-conotoxins, μ-KIIIA acts by blocking the ion conduction pore of the sodium channel, in contrast to μO-conotoxins such as MrVIA and MrVIB, which inhibit sodium channels by acting as gating modifiers but are also analgesic in a variety of animal models of pain [50]. Structure-activity studies with μ-KIIIA identified that five amino acid residues are important for sodium channel activity [51] and selectivity [52]. Furthermore, the solution structure reveals that four of the key residues are located on an α-helical region of the peptide [53,54]. With this important structure-function information, non-peptidic scaffolds were designed to mimic key amino acid side chains. In the first instance, a diketopiperazine (DKP) carboxamide **16** was designed to mimic the C_α_–C_β_ bond vectors of Lys7, Trp8 and His12 (Figure 7) [55].

Key elements in the scaffold design included appending a carboxamide to the DKP core, thereby stabilizing an internal hydrogen bond. According to *in silico* modeling, this conformation would be required in order to correctly mimic the His12 side chain. The presence of the hydrogen bond was evidenced by the downfield NMR resonance of the carboxamide NH (~10 ppm) in deuterated DMSO, suggesting that this essential conformation should be adopted under assay conditions [56]. The μ-KIIIA mimetic **16** was evaluated in a patch-clamp assay and found to weakly block Na_V_1.7 (20% at 100 μM). An important consideration for this molecule is the orientation of the Trp side chain mimic. It has been well established that aromatic side chains attached to a DKP will favor an orientation in which the ring is folded over the DKP ring, stabilized by π-stacking interactions [57,58]. The energy difference of ~3 kcal/mol between a folded conformation and one in which the ring is extended away from the core, potentially translates to a 100-fold loss in activity [59]. This could explain the weak activity if the indole ring needs to be extended to mimic μ-KIIIA, and indeed this is a focus of further optimization.

**Figure 8:**
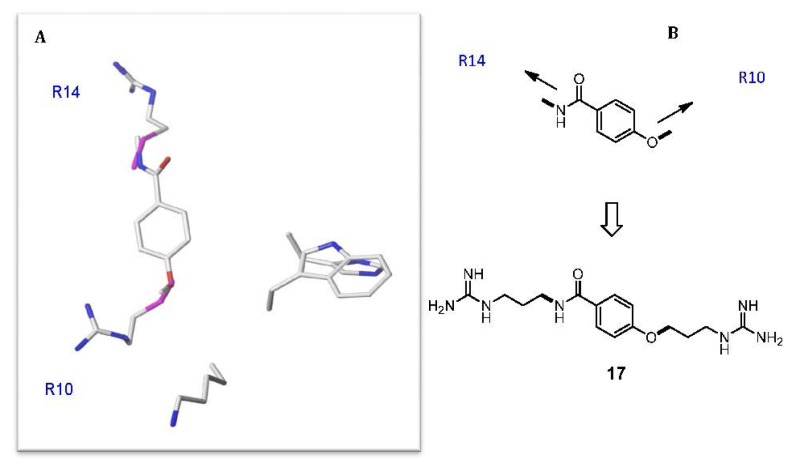
(**A**) *In silico* de novo designed μ-KIIIA peptidomimetic. A benzamide scaffold was designed to reproduce the C_α_–C_β_ bond vectors of the Arg14 and Arg10 residues. (**B**) The functionalized molecule **17** incorporating the terminal guanidine moieties.

A second peptidomimetic scaffold, **17**, was designed to probe the importance of the arginine side chains in μ-KIIIA [60]. A relatively simple benzamide mimicked the C_α_–C_β_ bond vectors of Arg10 and Arg14. The mimetic **17** (Figure 8) was found to weakly block Na_V_1.7 in patch clamp assays (20% at 100 μM). Although the activity of **17** is weak, it potentially represents a useful platform for medicinal chemistry optimization owing to its favorable molecular weight (335 g/mol) and synthetic tractability.

The current landscape with respect to mimicking the conotoxins with small organic molecules clearly highlights the challenges associated with this approach. In the examples reviewed here, the peptidomimetics are substantially less potent than the parent peptide. In each case, the authors were no longer guided by the initial design and the assumptions of mimicking the three residues. However, such compounds can be regarded as “hits”, where medicinal chemistry optimization may be expected to lead to improved potency. This serves as a notable advantage over the endogenous peptide, where generating SAR through analogues is not as efficient, and potentially allows one to bridge the gap in activity between the peptide and the mimetic, but in a more drug-like small molecule.

## 3. Modified Peptides

### 3.1. Truncated Conotoxins

An alternative to the peptidomimetic approach is to alter the peptide to enhance its drug-like properties and maintain biological activity. For many of the conotoxins, biological activity can be attributed to a relatively small fraction of the total number of amino acid residues, suggesting that the native peptide may be truncated without perturbing activity. Such analogues present a more drug-like and economical starting point for advancing towards novel therapeutics. In a study by Jin *et al.*, insights into the structure-function relationships of α-conotoxin PnIA were revealed through systematic truncation of the two disulfide loops [61]. The α-conotoxins inhibit nicotinic acetylcholine receptors (nAChR) by binding at the subunit interfaces of the extracellular domains of these pentameric ion channels [5,62]. Structurally, α-PnIA is characterized by two loops containing four and seven residues in the first and second loop, respectively (4/7 framework) [63]. Excising one to three amino acids from the second loop resulted in gradual loss of secondary structure and stability, yet the peptide retained potent affinity for the α7 nicotinic acetylcholine receptor (α7 nAChR). Truncation of four amino acids from the second loop, however, adversely affected both stability and activity.

Since truncated peptides are typically unstructured, the introduction of lactam bridges is an effective tool to stabilize α-helical conformations [64,65,66,67,68,69]. Truncated analogues of μ-KIIIA stabilized with lactam bridges were explored by Khoo *et al.* [70]. Their strategy involved removal of disulfide bridges along with residues at both the *N*- and *C*-termini of the native peptide, affording a smaller and thus more readily optimisable peptide. The α-helix was stabilized by incorporating an *i* to *i* + 4 lactam bridge between Lys and Asp residues across three positions in μ-KIIIA: Residues 5–9, 7–11 and 9–13. It was found that the position of the lactam bridge did not affect the helical propensity of the truncated analogues but it was important for VGSC activity. In particular, the lactam bridge between Residues 7 and 11 significantly reduced the peptide’s ability to block VGSCs. This was consistent with previous studies, which had shown that Lys7 and Asp11 were essential for activity [71]. The most active analogue was achieved by linking Residues 9 and 13, yielding an IC_50_ of 13.3 μM against Na_V_1.2; this is, however, still significantly less potent than the native peptide (IC_50_ 0.061 μM).

Stevens and co-workers utilized the previously reported structure-function characteristics of the μ-conotoxins KIIIA and BuIIIC [72] to design novel truncated analogues [73]. The initial truncated analogue comprised a first intercysteine loop based on μ-KIIIA and a second loop emulating that in μ-BuIIIC. The third loop between Cys1 and Cys9 was removed. The resultant 13-residue derivative displayed moderate block of VGSCs and was selective for the Na_V_1.2 isoform (63% at 20 μM). Ser4 was removed and an Ala residue was introduced into the truncated peptide at the position initially occupied by Cys9. The authors reasoned that this addition would restore the appropriate spacing required for the peptide to adopt an α-helix. Although a helical conformation was not observed for this analogue, it was found to be a potent blocker of Na_V_1.2 (IC_50_ 78 nM). Its potency was further improved by substituting His10 with an Arg residue, resulting in an IC_50_ for Na_V_1.2 of 34 nM.

### 3.2. Disulfide Isosteres

**Figure 9:**
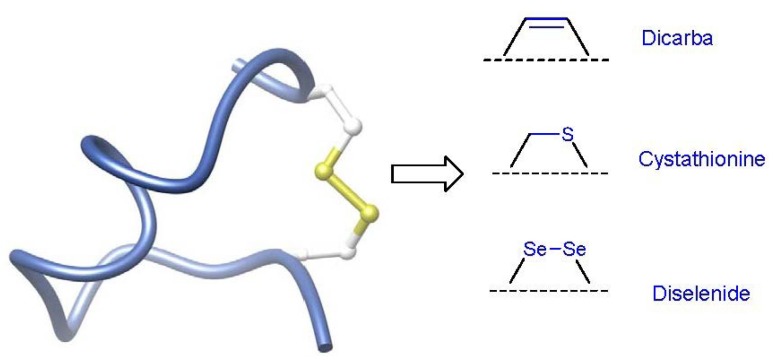
Strategies used to replace the disulfide linkage in conotoxins with stable isosteres. Diselenide, dicarba and cystathionine bridges have proven most effective. In addition, the dicarba bridge can also be reduced to a methylene or oxidized to an alkyne bridge.

An intrinsic feature of the conotoxins is the presence of disulfide bonds, which are thought to be critical for structure and function [74]. However, these disulfides are also a potential metabolic liability as it has been shown that they are susceptible to reduction in certain extracellular environments such as the blood [75]. An attractive strategy to overcome this limitation is their replacement with more stable alternatives. To this end diseleno [76], dicarba [77] and thioether [78,79,80] linkers have been incorporated in place of native cysteine bonds in a number of disulfide-containing bioactive peptides (Figure 9). The dicarba approach in particular has emerged as an effective and widely used tool due in large part to the development of Grubbs olefin metathesis [81,82,83,84,85,86,87].

Compared with the sulfur-sulfur bond in disulfide bridges (2.02 Å), selenium-selenium bond lengths in diselenium bridges are slightly longer (*ca* 2.05 Å), while the carbon-carbon bond lengths in dicarba bridges are shorter (*ca.* 1.34 Å) [88]. Although these atomic distances do result in stereochemical variations proximal to the introduced bridge, this has not been shown to significantly affect the overall structure, as evidenced by several examples where biological function has been maintained [89,90]. A further advantage of disulfide isosteres is their increased stability, resulting in improved pharmacokinetic properties [91,92]. The cystathionine thioether, where one of the sulfur atoms of a disulfide bond is substituted with a methylene group, should more closely approximate the geometry of cystine than dicarba or lanthionine analogues and is therefore expected to cause minimal structural perturbations [93].

The strategy of incorporating disulfide isosteres has been applied to several conotoxins. α-ImI, a selective nAChR antagonist, is of considerable interest as a biological tool and as a lead for potentially developing novel therapeutics [5,23]. α-ImI is a 12-residue peptide with two disulfide bridges linking Residues 2 and 8 and 3 and 12, respectively [94]. Diselenide linkages were incorporated into α-ImI in work reported by Armishaw *et al.* [76]. These bridges were incorporated systematically; the first analogue contained a bridge only between Residues 2 and 8, the second contained a bridge only between Residues 3 and 12, and in the third both disulfides were replaced with diselenide bridges. Importantly, the conformations of the three selenocysteine-containing isomers were in good agreement with that of the native structure as determined by NMR and CD spectroscopy. Furthermore, all three isomers possessed similar bioactivity (IC_50_ ~50 nM) at the α7 nAChR, comparable to the native peptide (IC_50_ 69 nM). When exposed to glutathione and human mercaptalbumin, no scrambling of the selenocysteine framework was observed, in contrast to native α-ImI, which was completely scrambled under these conditions. 

The dicarba approach to replacing the disulfide bonds was also applied to α-ImI by MacRaild *et al.* [95] Analogues containing single carbon-carbon bonds in place of either the 2 to 8 (isomer 1) or 3 to 12 (isomer 2) disulfide bridges had solution conformations similar to that of native α-ImI. The ability of the isomers to antagonize α7 nAChR was determined by catechol release after nicotine stimulation of cultures of bovine adrenal chromaffin cells. Both Isomers 1 and 2 were found to inhibit adrenaline release with similar potency, with IC_50_ values of 10 and 15 μM, respectively. Additionally, the effect of the dicarba analogues on ACh-evoked ion currents in frog oocytes expressing α7 nAChR was determined. At 2.5 μM, Isomer 1 inhibited response by 60% in the presence of ACh (300 μM), which was comparable to α-ImI (68%). On the other hand, Isomer 2 was inactive at 2.5 μM. The stability of the dicarba-ImI isomers in plasma was enhanced significantly [96]. Very recently, dicarba bridges have also been introduced into Vc1.1, producing some interesting changes in target specificity; the 2,8-dicarba Vc1.1 isomer retained activity at GABA_B_ receptors, whereas the isomeric 3,16-dicarba Vc1.1 peptide retained activity at the α9α10 nAChR [97].

**Figure 10:**
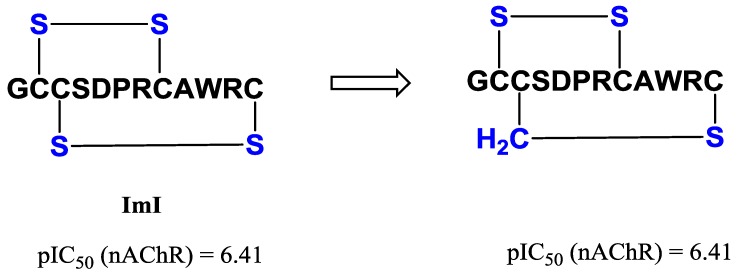
α-conotoxin ImI has two disulfide bridges linking Residues 2–8 and 3–12, respectively [98]. Replacement of the disulfide bond in the second intercysteine loop of α-ImI with a cystathionine, resulted in retention of structure and against α7 nAChR.

A study by Dekan *et al.*, exemplifies the use of cystathionine as a replacement for the disulfide bond in α-ImI [98]. The strategy involved regioselective formation of cystathionine bonds in the first and second intercysteine loops as well as a dual cystathionine analogue. Structurally all three analogues were identical to α-ImI, as determined by two-dimensional ^1^H NMR. However, only the second loop thioether analogue retained significant activity against α7 nAChR, being equipotent with α-ImI (Figure 10, pIC_50_ 6.41).

### 3.3. Cyclization Strategies

Since conformation is known to play a critical role in bioactivity and bioavailability, cyclization can be used to stabilize the conformation of the peptide [99,100,101]. Such modifications can induce stability and more favorable pharmacokinetic properties as evidenced by the number of cyclic peptides in pre-clinical evaluation or that have advanced to use in humans [102,103,104,105]. The *N*-*C* terminal cyclization strategy has been applied successfully to several conotoxins, as reviewed by Clark *et al*. [106]. A pertinent example is the application of a backbone macrocyclization strategy to Vc1.1 [107]. A member of the α-conotoxin family, Vc1.1 is a 16-residue peptide with a short internal α-helix and two disulfide bridges [108]. It is a potent analgesic [109] that binds to α9,α10 nACh receptors [110,111] but can also interact with GABA_B_ receptors [112]; indeed, the latter may be the key target for its analgesic activity as it has been shown that Vc1.1 can inhibit high voltage-activated calcium channel currents in dorsal root ganglion (DRG) neurons via GABA_B_ receptor-mediated inhibition of N-type (Ca_V_2.2) calcium channels [107,113,114]. The design of the cyclic α-conotoxin was based on the three-dimensional structure of Vc1.1 [108]. A six-residue linker composed of Gly and Ala was used to span the 12 Å distance between the *N*- and *C*-termini. This cyclic analogue was more potent than linear Vc1.1 as a GABA_B_-mediated calcium channel blocker and displayed improved stability relative to linear Vc1.1. When exposed to simulated intestinal fluid and human serum, only minor disulfide rearrangement was observed; by contrast, linear Vc1.1 was found to undergo significant disulfide rearrangement (42% in simulated intestinal fluid and 46% in human serum) to inactive isomers. Importantly, cyclic Vc1.1 showed dose-dependent relief of neuropathic pain in rats when administered orally. Its activity at 1.3 mg/kg was comparable to that of gabapentin, a commonly prescribed oral analgesic, at 30 µg/kg, highlighting the potency and bioavailability of the cyclic conotoxin [107].

### 3.4. Backbone Prosthesis

In an approach termed “backbone prosthesis” [115], μ-conotoxin analogues were designed in which nonessential peptidic regions were replaced by non-peptidic spacers. Referred to as “polytides” [115], these analogues can display improved pharmacological properties, and in many cases retained or even improved biological activity. Typical spacers that have been used for improving the therapeutic properties of peptides and proteins include polyethylene glycol (PEG) [116], 6-aminohexanoic acid [117] and amino-3-oxapentanoic acid [118,119].

The concept of backbone prosthesis was first applied to μ-SIIIA [120], a potent blocker of VGSCs, by Bulaj and co-workers [115]. Previously described structure-activity relationships suggested that neither the length of the first cysteine loop nor the *N*-terminal residue was critical for blocking VGSCs [49]. Thus, the “nonessential” *N*-terminal residue and the two Gly residues in the first loop were replaced with non-peptide spacers. Two commonly used backbone spacers were employed; in the first analogue, both the *N*-terminus and the Gly-Gly-fragment were replaced with amino-3-oxapentanoic acid (PEG-SIIIA). In the second, 6-aminohexanoic acid (AHX-SIIIA) was used in place of amino-3-oxapentanoic acid. Whilst there was some structural variation between μ-SIIIA and the prosthesis-containing analogues, the region *C*-terminal to the backbone replacement displayed a similar conformation to native μ-SIIIA. Importantly, the residues critical to activity, Lys11, Trp12, and His16, adopted the same spatial arrangement in all three structures. The polytides displayed impressive sodium channel activity: After 20 min of exposure to 5 μM concentrations the polytides were able to block ~45%–55% of the sodium channel current, greater than the 20% block exhibited by μ-SIIIA. Increasing the concentration to 25 μM resulted in 65% inhibition for μ-SIIIA and 80% inhibition for the polytides. In addition, both polytides were shown to possess analgesic activity in the inflammatory pain assay in mice [121], with PEG-μ-SIIIA being even more potent than the endogenous peptide. All three peptides were analgesic at doses of 10 nmol per animal, although both polytides were more active in the Phase II response than μ-SIIIA. At doses of 10 nmol per animal, PEG-μ-SIIIA exhibited more pronounced and longer-lasting analgesic activity in the inflammatory phases.

The backbone prosthesis approach has been extended to μ-KIIIA, where 5-amino-3-oxapentanoic acid (Aopn), was used to replace two nonessential Ser residues in disulfide-deficient μ-KIIIA analogues [122]. Key to their approach was to identify which of the three disulfide loops in μ-KIIIA could be removed without compromising biological activity. The authors observed that removal of the first Cys1–Cys9 disulfide bridge had minimal effect on binding to Na_V_1.2, with this analogue being almost equipotent with μ-KIIIA [53,54]. The Cys2A–Cys15A deletion analogue was less potent, with a *K*_d_ for Na_V_1.2 of 170 nM. On the other hand, the analogue devoid of the Cys4–Cys16 bridge was essentially inactive. The Aopn spacer was then used to replace two adjacent Ser residues (Ser5–Ser6) in the disulfide deletion analogue since alanine scanning had shown that these residues had little effect on binding. Furthermore, the authors reasoned that Ala1 in the Cys1–Cys9 analogue seemed unlikely to play a major role in the interactions with the sodium channels, and thus removed this residue from the Aopn containing peptoid. This minimized analogue was able to block Na_V_1.2 with a *K*_d_ of 46 nM and was analgesic in the inflammatory pain assay in mice.

## 4. Conclusions

Conotoxins have been shown to be potent inhibitors of a broad range of ion channels and receptors, many of which have been identified as drug targets. As such, the conotoxins have great potential to serve as leads for the next generation of drugs to treat conditions where there are clearly unmet clinical needs. The challenge remains to translate the bioactivity of the conotoxins into therapeutically relevant molecules. The last decade has seen a number of novel strategies applied to overcoming the inherent limitation of peptides as drugs. To date, the peptidomimetic strategy has proved challenging, with attempts at mimicking the conotoxins with non-peptide molecules generally resulting in significant losses in potency. A potential advantage of the mimetic approach is that the molecules generated lend themselves to medicinal chemistry optimization. On the other hand, strategies to directly modify the peptide toxins have proven to be quite effective. In particular, the dicarba and cyclization approaches have produced potent and more stable analogues of the native peptides and these approaches could see more conotoxins progress towards the clinic.
